# The cAMP-PKA Signaling Pathway Regulates Pathogenicity, Hyphal Growth, Appressorial Formation, Conidiation, and Stress Tolerance in *Colletotrichum higginsianum*

**DOI:** 10.3389/fmicb.2017.01416

**Published:** 2017-07-25

**Authors:** Wenjun Zhu, Man Zhou, Zeyang Xiong, Fang Peng, Wei Wei

**Affiliations:** ^1^College of Biology and Pharmaceutical Engineering, Wuhan Polytechnic University Wuhan, China; ^2^Institute for Interdisciplinary Research, Jianghan University Wuhan, China

**Keywords:** *Colletotrichum higginsianum*, cAMP-PKA signaling pathway, ChPKA1, pathogenicity, appressorial formation, stress tolerance

## Abstract

*Colletotrichum higginsianum* is an economically important pathogen that causes anthracnose disease in a wide range of cruciferous crops. Understanding the mechanisms of the cruciferous plant–*C. higginsianum* interactions will be important in facilitating efficient control of anthracnose diseases. The cAMP-PKA signaling pathway plays important roles in diverse physiological processes of multiple pathogens. *C. higginsianum* contains two genes, *ChPKA1* and *ChPKA2*, that encode the catalytic subunits of cyclic AMP (cAMP)-dependent protein kinase A (PKA). To analyze the role of cAMP signaling pathway in pathogenicity and development in *C. higginsianum*, we characterized *ChPKA1* and *ChPKA2* genes, and adenylate cyclase (*ChAC*) gene. The *ChPKA1* and *ChAC* deletion mutants were unable to cause disease and significantly reduced in hyphal growth, tolerance to cell wall inhibitors, conidiation, and appressorial formation with abnormal germ tubes, but they had an increased tolerance to elevated temperatures and exogenous H_2_O_2_. In contrast, the *ChPKA2* mutant had no detectable alteration of phenotypes, suggesting that *ChPKA1* contributes mainly to PKA activities in *C. higginsianum*. Moreover, we failed to generate Δ*ChPKA1ChPKA2* double mutant, indicating that deletion of both PKA catalytic subunits is lethal in *C. higginsianum* and the two catalytic subunits possibly have overlapping functions. These results indicated that ChPKA1 is the major PKA catalytic subunit in cAMP-PKA signaling pathway and plays significant roles in hyphal growth, pathogenicity, appressorial formation, conidiation, and stress tolerance in *C. higginsianum*.

## Introduction

The hemibiotrophic fungus *Colletotrichum higginsianum* is an economically important pathogen that causes anthracnose disease on a wide range of cruciferous plants, including *Brassica, Raphanus*, and *Arabidopsis thaliana* (Narusaka et al., 2004; Yang et al., 2008; Hyde et al., 2009). To invade plant tissue successfully, *C. higginsianum* conidia germinate and form the melanized infection structure appressorium at the tips of conidial germ tubes after attaching to plant surfaces. After that, *C. higginsianum* penetrates the cuticle and plant cell wall using high turgor pressure generated in melanized appressoria for further infection (O'Connell et al., 2012). The morphogenesis and invasion of several fungal pathogens are dependent on recognition and transduction of host environmental stimuli and surface cues to its downstream via multiple signal transduction pathways, especially the cyclic adenosine monophosphate (cAMP)-PKA signaling pathway (Kronstad et al., 2011; Li et al., 2012). Protein kinase A (PKA) is a serine/threonine protein kinase that serves as the main intracellular target of cAMP, a key secondary messenger synthesized by adenylate cyclase (AC). PKA consists of two catalytic and two regulatory subunits; binding of cAMP to regulatory subunits leads to the activation of catalytic subunits, which phosphorylate downstream target proteins and regulate a variety of physiological processes (Robertson and Fink, 1998).

To date, the cAMP-PKA signaling pathway is known to function in a range of physiological processes in fungi, including growth, cell wall integrity, cell differentiation, stress responses, pathogenicity, colonization, and secondary metabolism (Lengeler et al., 2000; Fillinger et al., 2002; Oliver et al., 2002; Xue et al., 2008; Turrà et al., 2014). In soilborne fungal pathogen *Fusarium oxysporum*, deletion of cAMP-dependent PKA (*FoCPKA*) led to failure to penetrate into the vascular system of *A. thaliana* roots, loss of virulence, and reduced vegetative growth and spore production (Kim et al., 2011). In *F. verticillioides*, the cAMP-PKA pathway is involved in mycelia growth, conidiation, bikaverin production, and plant infection (Choi and Xu, 2010). In *F. graminearum*, functional studies of the *FAC1* (adenylate cyclase), *CPK1* and *CPK2* genes have demonstrated that the cAMP-PKA signaling pathway plays significant roles in morphogenetic switch, growth, deoxynivalenol (DON) production, pathogenicity, and sexual reproduction (Bormann et al., 2014; Hu et al., 2014). In *Ustilago maydis*, the cAMP-PKA signaling pathway is important for dimorphic switching and mating (Durrenberger et al., 1998). In *Magnaporthe oryzae*, the cAMP-PKA signaling pathway is involved in surface recognition, asexual, and pathogenic differentiation (Ramanujam and Naqvi, 2010). The Cyclase-associated protein Cap1 from *M. oryzae* is involved in activation of adenylate cyclase, appressorium morphogenesis, and plant infection (Zhou et al., 2012). Deletion of PKA catalytic subunit gene *Cpk1* and adenylate cyclase gene *CAC1* in *C. lagenarium* caused defect in conidia germination and pathogenicity, attenuated growth rate and reduced conidiation (Yamauchi et al., 2004). In *C. orbiculare*, Ras GTPase activating protein CoIra1 contributes to infection-related morphogenesis by regulating cAMP and MAPK signaling pathways (Harata and Kubo, 2014). The AreA transcription factor from *F. graminearum* was shown to mediate the regulation of DON synthesis by cAMP signaling (Hou et al., 2015). Recently, it was reported that cAMP signaling pathway is involved in the regulation of DON biosynthesis by two pathway-specific transcription factors TRI (trichothecene biosynthesis) 6 and TRI10 (Jiang et al., 2016). Therefore, these findings suggest significant roles for cAMP-PKA signaling pathway in multiple physiological processes of different microorganisms, especially during the steps of surface recognition and penetration which are critical in the infection cycle of many plant pathogenic fungi. Inhibition on the cAMP-PKA signaling pathway of pathogens will disturb infection progress and facilitate efficient control of crop diseases.

Although many studies have been done to elaborate cAMP-PKA signaling pathway in other fungi, the specific roles of cAMP-PKA signaling for infection-related morphogenesis and infectious growth remain largely unknown in *C. higginsianum*. Because the cAMP-PKA signaling pathway contributes to multiple physiological processes of fungal pathogens, especially surface recognition and penetration (Li et al., 2012), characterization of *C. higginsianum* cAMP-PKA signaling will help elucidate the mechanism of the *C. higginsianum*-cruciferous crops interaction and facilitate the efficient control of anthracnose disease.

In this study, to better understand the cAMP-PKA signaling pathway in *C. higginsianum*, we investigated the functions of PKA catalytic subunits ChPKA1 (CH063_00098) and ChPKA2 (CH063_12956), and adenylate cyclase ChAC (CH063_06008). Whereas, Δ*ChPKA2* mutant had no obviously detectable phenotypes, the Δ*ChPKA1* and Δ*ChAC* mutants had similar phenotypes with pleiotropic defects in hyphal growth, appressorial formation, stress tolerance, conidiation, and pathogenicity. Application of exogenous cAMP could partially rescue the phenotype defects, indicating that ChPKA1 is the major PKA catalytic subunit in *C. higginsianum*. Moreover, although the conidia of Δ*ChPKA1* and Δ*ChAC* mutants had defects in appressorial formation at high conidial density (10^6^ conidia/ml), they partially differentiated into appressoria at low conidial density (10^4^ conidia/ml) with much longer germ tubes compared with that of wild type strain. In addition, we failed to obtain Δ*ChPKA1* Δ*ChPKA2* double mutant, indicating that deletion of both PKA catalytic subunit genes is lethal in *C. higginsianum*. Overall, our data indicate that cAMP-PKA signaling pathway plays essential roles in hyphal growth, stress tolerance, conidiation, appressorial formation, and pathogenicity in *C. higginsianum*.

## Materials and methods

### Strains and plant materials

*C. higginsianum* wild-type strain IMI349061 (CH-1) was cultured on potato dextrose agar (PDA) at 25°C and stored in PDA slants at 4°C for further use. The wild type plants of *A. thaliana* Col-0 used in this study for pathogenicity test were grown in greenhouse at 20 ± 2°C, under a 12 h light/dark cycle. *Agrobacterium tumefaciems* strain EHA105 was used for transformation of *C. higginsianum* and *Escherichia coli* strain JM109 was used to propagate all plasmids.

### Bioinformatics data and programs used in this study

The publicly available genomic sequence database of *C. higginsianum* at JGI (http://genome.jgi.doe.gov/Colhi1/Colhi1.home.html) was used to characterize *ChPKA1* (*CH063_00098*), *ChPKA2* (*CH063_12956*), and *ChAC* (*CH063_06008*) genes. BLAST analysis was done by using *NCBI* (http://www.ncbi.nlm.nih.gov/) and *UniProt* (http://www.uniprot.org/blast/). *MEGA* program was used for the production of phylogenetic tree with unrooted neighbor-joining method. Domains identification was performed by using *SMART MODE* (http://smart.embl-heidelberg.de/smart/change_mode.pl). The *Clustal X* program was used for amino acid alignments.

### Manipulation of nucleic acids

The genomic DNA of *C. higginsianum* wild type strain and other derivative transformants were extracted using genomic DNA purification kit (Axygen, USA) according to the manufacturer's protocols. Southern blot analysis was performed as previously described (Liu et al., 2013). The 20 μg genomic DNA of each strain was digested overnight, then size-fractionated through a 0.8% agarose gel and mounted on positively charged nylon membrane. The nylon membrane was then hybridized with a probe amplified by primers (Supplementary Table S1) and labeled with digoxigenin (DIG)-dUTP using the PCR DIG Probe Synthesis Kit (Roche, Mannheim, Germany) according to the manufacturer's protocol.

Total RNA was isolated with TriZOL reagent (Invitrogen, Carlsbad, USA) according to manufacturer's instructions and then stored at −80°C for further studies. The total RNA samples were treated with DNase I (RNase Free) (Takara, Dalian, China) at 37°C for 30 min and used to generate the first strand cDNA with the High Capacity cDNA Reverse Transcription Kit (Applied Biosystems™, Foster, CA, USA) according to manufacturer's instructions. The first strand cDNA was stored at −20°C for further studies. Genes expression was analyzed by quantitative real-time reverse polymerase chain reaction (qRT-PCR) using a Bio-Rad CFX96 (Bio-Rad, California, USA) and SYBR Premix Ex Taq II (TAKARA, Dalian, China), according to the manufacturer's instructions. The *C. higginsianum* β*-tubulin* gene (*CH063_04743*) was used to normalize the RNA sample for each round of qRT-PCR. The PCR conditions were as follows: denaturation at 95°C for 3 min; 40 cycles of 95°C for 15 s, 55°C for 20 s and 72°C for 25 s; final step of 72°C for 10 min. Primer pairs for qRT-PCR reactions were designed either across or flanking an intron and listed in Supplementary Table S1. For each gene, qRT-PCR assays were repeated at least twice, with each repetition having three independent replicates.

### Gene replacement and complementation

To characterize *ChPKA1, ChPKA2*, and *ChAC* genes, the genes replacement vectors pChPKA1-3300, pChPKA2-3300, and pChAC-3300 were generated as described (Ma et al., 2017). The 5′(about 500 bp)- and 3′(about 500 bp)- flanks of the ORF of each genes were amplified from genomic DNA of the wild type strain CH-1 by PCR with primer pairs (Supplementary Table S1). The 5′- and 3′- fragments of each genes were then cloned into the upstream and downstream of *hph* cassette respectively, using Gibson Assembly Master Mix kit (New England Biolabs, Massachusetts, USA) according to the manufacturer's instructions. Then, the 5′ fragment-*hph*-3′ fragment cassettes of each gene were cloned into pNeoP3300 (Wei et al., 2016), resulting in gene replacement vectors, which had the selective marker *hph* gene flanked by the ORF flanking sequences from each of the genes (Supplementary Figure S1A).

The gene complementation vectors pChPKA1-Com, pChPKA2-Com and pChAC-Com were constructed that the cDNA of each gene was amplified by RT-PCR with primers (Supplementary Table S1) and cloned into the same sites of pCIT vector, which contained the constitutive P*trpC* promoter and T*trpC* terminator. Finally, the P*trpC*-cDNA-T*trpC* cassette was cloned into pNeoP3300, resulting in complementation vector.

*Agrobacterium*-mediated transformation was performed as previously described (Wei et al., 2016). Transformants were transferred to PDA plates containing 50 μg/ml of hygromycin B (Roche, Mannheim, Germany) or 150 μg/ml G418 (Ameresco, OH, USA) for a second round of selection and further confirmed by Southern blot and qRT-PCR analysis.

### Phenotype analysis

The growth rates of all the transformants and the wild-type strain were assayed as previously described (Wei et al., 2016). All strains were initially grown on PDA for 7 days. The mycelial agar discs were then taken from the active colony edge, inoculated into the center of the PDA petri dish at 25°C, and then the colony diameters from 1 to 7 dpi were examined. The colony morphology and conidiation of all strains were examined after being grown on PDA plates at 25°C for 14 and 7 days, respectively.

Pathogenicity assays of *C. higginsianum* on Arabidopsis were performed as previously described (Yuan et al., 2016). The conidia harvested from 5-days-old PDA agar cultures at 25°C were washed with sterile distilled water twice. The leaf surfaces of each pot of 4-weeks-old Arabidopsis were sprayed with 1 ml conidial suspensions (10^6^ conidia/ml). The infected Arabidopsis plants were incubated in humid chambers at 25°C for 6 days, and the symptoms were then observed. The experiment was repeated three times, and each strain infected three *A. thaliana* plants each time.

Appressorial formation was measured. 10 μl conidial suspensions (10^6^ conidia/ml for high conidial density and 10^4^ conidia/ml for low conidial density) were spotted on hydrophobic coverslips and incubated in darkness at 25°C for 24 h. The percentage of appressorial formation was determined by microscopic examination for at least 200 conidia or appressoria using Nikon Eclipse 80i microscope (Nikon, Tokyo, Japan), under bright-field model using 40 × fold magnification. Each test was repeated at least three times.

The tolerance of transformants and wild-type strain to multiple stress was performed. The mycelial plugs from the active colony edge were inoculated on PDA containing 300 μg/ml Calcofluor White (CFW), 10 mM H_2_O_2_, and 300 μg/ml Congo Red (CR) respectively and PDA media as controls. The growth inhibition rate (%) of stress to the transformants and wild type strain was analyzed by measuring the colony diameters as previously described (Wei et al., 2016).

The tolerance of these strains to high temperature was performed by inoculating mycelial plugs from the active colony edge of each strain on PDA and then culturing at 25 and 35°C respectively for 6 days. The phenotypes and growth inhibition rate of these strains were then analyzed.

### Statistical analysis

The data were analyzed with Origin 7.5 (OriginLab Corporation, Massachusetts, USA) using ANOVA (one-way, *P* ≤ 0.01). Results of all graphs represent the mean value ± SD. Asterisks and different letters in the graphs indicate statistical differences, *P* ≤ 0.01.

## Results

### Bioinformatics analysis of ChPKA1 and ChPKA2

The *C. higginsianum* genome contains two PKA catalytic subunit genes, CH063_00098 and CH063_12956, that were respectively named *ChPKA1* and *ChPKA2. ChPKA1* encodes a 507-amino-acids protein and *ChPKA2* encodes a 392-amino-acids protein. The *SMART MODE* (http://smart.embl-heidelberg.de/smart/change_mode.pl) analysis result indicates that both of ChPKA1 and ChPKA2 contain a Serine/Threonine protein kinase catalytic (S_TKc) domain and an Extension to Ser/Thr-type protein kinase (S_TK_X) domain (Supplementary Figure S2A), which plays a key role in catalysis of protein phosphorylation. BLAST searches of fungal genomes with ChPKA1 and ChPKA2 showed the presence of homologs in a large number of fungi. Multiple sequence alignment and phylogenetic analysis revealed significant sequence conservation (Supplementary Figures S2B–D).

### Up-regulation of *ChPKA1* and *ChAC* expression but not *ChPKA2* during appressorial formation

**Figure 1:**
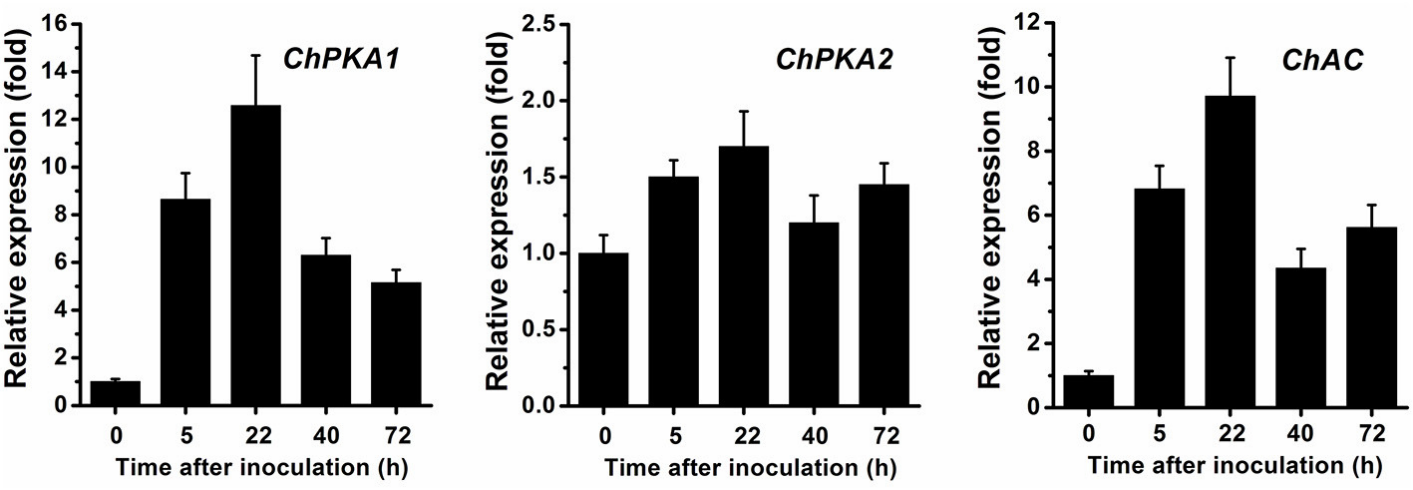
Expression patterns of *ChPKA1, ChPKA2*, and *ChAC* during infection stages were evaluated by qRT-PCR. RNA was extracted from infected Arabidopsis seedlings at 0 hpi, 5 hpi (conidial germination), 22 hpi (appressorial formation), 40 hpi (biotrophic infection stage), and 72 hpi (necrotrophic infection stage), respectively. The genes expression of *C. higginsianum* conidia inoculated on Arabidopsis at 0 h was set as level 1 and relative levels of transcript were calculated using the comparative Ct method. Transcript level of the *C. higginsianum* β*-tubulin* gene (*CH063_04743*) was used to normalize different samples. Data represent means and standard deviations of three independent replications.

To gain insight into the functions of *ChPKA1, ChPKA2*, and *ChAC*, we firstly examined the gene expression patterns during different infection stages of *C. higginsianum* by qRT-PCR as previous described (Liu et al., 2013). The result demonstrated that the expression levels of *ChPKA1* and *ChAC* but not *ChPKA2*, were significantly up-regulated during conidia germinating (5 hpi) and appressorial formation (22 hpi) stages (Figure 1), indicating that ChPKA1 and ChAC may play significant roles in conidia germinating and appressorial formation of *C. higginsianum*.

### ChPKA1 and ChAC but not ChPKA2 are indispensable for growth, colony phenotype, and conidiation

**Figure 2:**
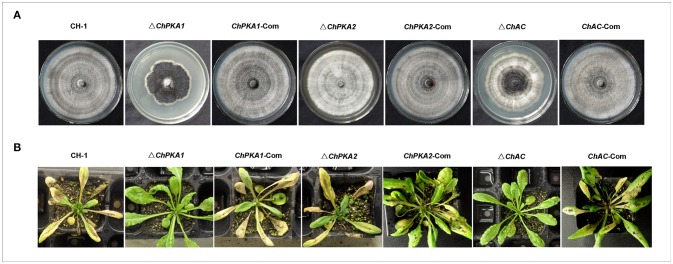
Phenotypes of wild type strain CH-1, *ChPKA1* deletion mutant Δ*ChPKA1, ChPKA2* deletion mutant Δ*ChPKA2, ChAC* deletion mutant Δ*ChAC*, and complementary strains *ChPKA1*-Com, *ChPKA2*-Com and *ChAC*-Com. **(A)** Colony morphology of indicated strains. These strains were grown on PDA medium at 25°C for 14 days. **(B)** Pathogenicity assays of indicated strains. 4-weeks-old *A. thaliana* were respectively sprayed with 1 ml conidial suspensions (10^6^ conidia/ml) of indicated strains and the infected Arabidopsis plants were incubated in darkness and humid chambers at 25°C for 6 days, and then the symptoms were observed.

**Figure 3:**
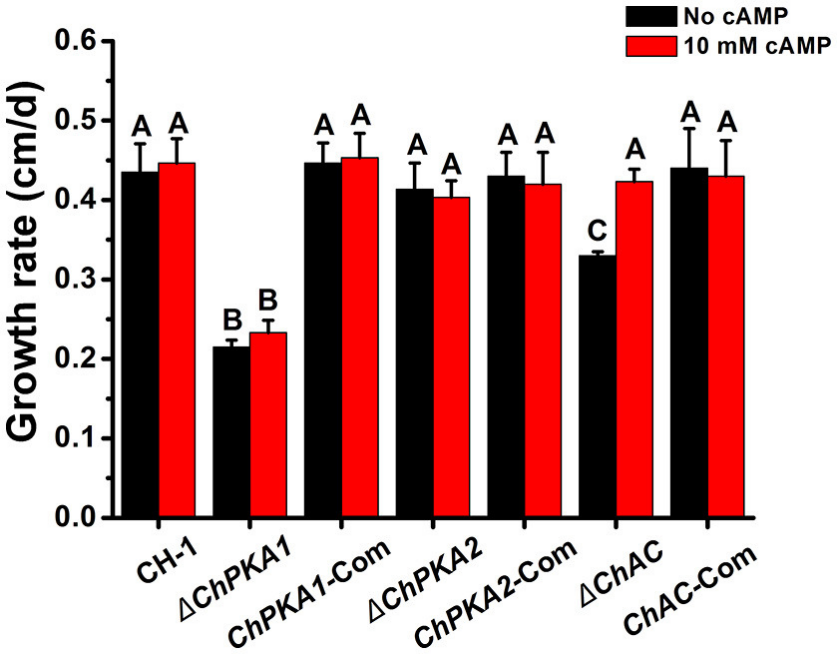
Growth rate of indicated strains. Growth rate of indicated strains cultured on PDA medium with (red) or without (black) exogenous 10 mM cAMP at 25°C. Bars represent standard deviations of three replications. Different letters in the graph indicate statistical differences, *P* ≤ 0.01.

**Figure 4:**
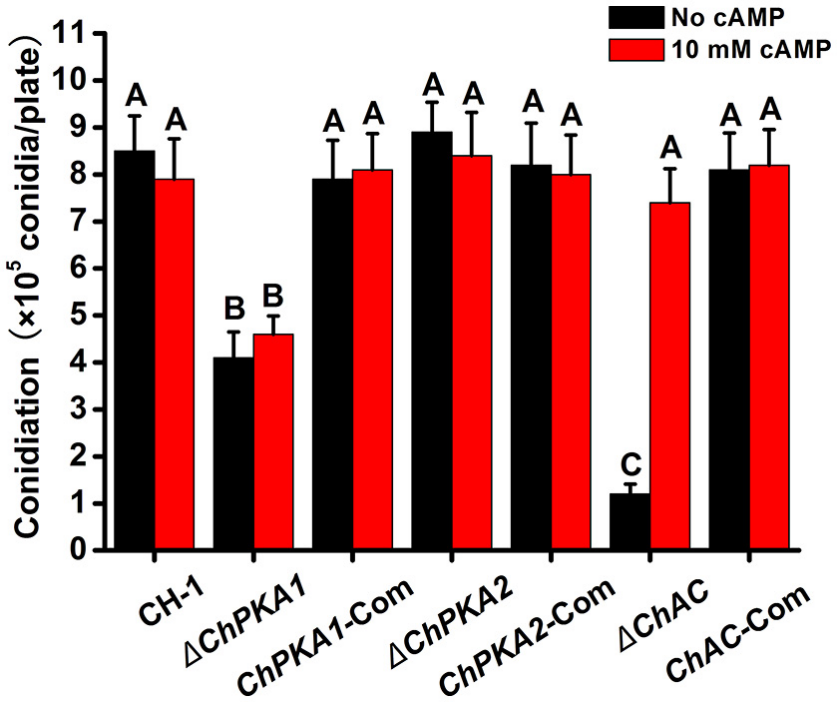
Conidial production of indicated strains. Conidiation of indicated strains cultured on PDA plates with (red) or without (black) exogenous 10 mM cAMP at 25°C for 7 days. Conidiation of each strain was determined by collecting conidia with a hematocytometer. Bars represent standard deviations of three replicates. Different letters in the graph indicate statistical differences, *P* ≤ 0.01.

To assess the function of ChPKA1 and ChPKA2 in *C. higginsianum*, the *ChPKA1* and *ChPKA2* deletion mutants were generated and screened by growing on PDA containing hygromycin and further confirmed by Southern blot and qRT-PCR (Supplementary Figures S1B,C). Whereas the *ChPKA2* deletion mutant Δ*ChPKA2* had no detectable phenotype variation compared with wild type strain, the *ChPKA1* deletion mutant Δ*ChPKA1* showed a significantly reduced growth rate (Figures 2A, 3; Supplementary Table S2), and the Δ*ChPKA1* sectored on PDA with obvious dark color and abnormal colony (Figure 2A). Compared with Δ*ChPKA2* and wild type strain, the Δ*ChPKA1* also showed obvious reduction of conidiation (Figure 4; Supplementary Table S2). For complementation assays, the complementary vector pChPKA1-Com, pChPKA2-Com, and pChAC-Com were reintroduced into mutants to generate complementary strains *ChPKA1*-Com, *ChPKA2*-Com, and *ChAC*-Com. Defects in hyphal growth, colony morphology, and conidiation were rescued in *ChPKA1*-Com and *ChAC*-Com strains (Figures 2A, 3, 4; Supplementary Table S2). These results indicated that deletion of *ChPKA1* but not *ChPKA2* is directly responsible for the phenotype defects in Δ*ChPKA1*, and ChPKA1 is involved in growth, colony phenotype and conidiation in *C. higginsianum*.

In addition, as adenylate cyclase (AC) contributes to the synthesis of cAMP and plays a significant role in cAMP-PKA signaling pathway, we also functionally analyzed ChAC by gene replacement of *ChAC* (CH063_06008) adenylate cyclase gene to generate *ChAC* knockout mutant Δ*ChAC* (Supplementary Figure S1). Compared to wild type strain, the growth rate and conidiation of Δ*ChAC* were obviously reduced on PDA medium (Figures 3, 4), and the Δ*ChAC* also showed an albino colony after 6 days incubation on PDA (**Figures 7A,B**). Furthermore, in the presence of exogenous 10 mM cAMP in PDA medium, the growth rate and conidiation in the Δ*ChAC*, but not Δ*ChPKA1*, mutant were restored to the levels of the wild type strain (Figures 3, 4). These results further suggested the importance of cAMP-PKA signaling pathway in the regulation of growth and conidiation in *C. higginsianum*.

Totally, we had obtained at least five individual mutant strains and three individual complementary strains for each gene, and all the mutants and complementary strains of each gene showed the same phenotype (Supplementary Table S2). Thus, we selected two mutants and one complementary strain of each gene for Southern blot, and choose one mutant and one complementary strain of each gene for further phenotype analysis.

### ChPKA1 and ChAC are important for pathogenicity and appressorial formation

To further assess the function of ChPKA1 in pathogenicity, the infection assays against the Arabidopsis were performed as described above. The results demonstrated that the wild type, complementary strains and Δ*ChPKA2* could cause typically water-soaked and dark necrotic lesions on leaves, whereas the Δ*ChPKA1* and Δ*ChAC* failed to cause obvious disease symptoms on *Arabidopsis* leaves after inoculating for 6 days (Figure 2B), which is similar to previous study in which the *PKA* and *AC* mutants of *C. lagenarium* did not cause any obvious lesions on cucumber leaves, even inoculated on wounded leaves (Yamauchi et al., 2004). These results indicated that *ChPKA1* and *ChAC* are required for pathogenicity in *C. higginsianum*.

**Figure 5:**
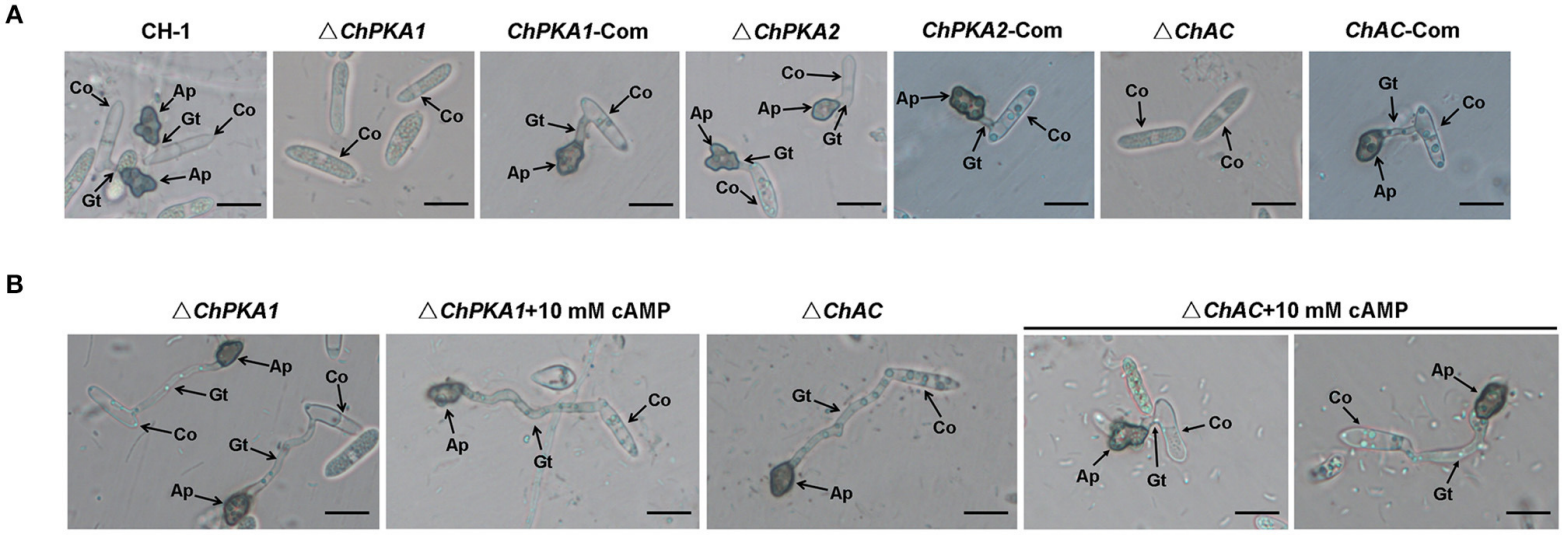
The appressorial development of indicated strains on hydrophobic coverslips. **(A)** The development of appressoria on plastic microscopic coverslips at 25°C for 24 h. Conidial density was 10^6^ conidia/ml. **(B)** Appressorial formation on plastic microscopic coverslips at 25°C for 24 h, with or without exogenous 10 mM cAMP. Conidial density was 10^4^ conidia/ml. The images were taken by using Nikon Eclipse 80i microscope (Nikon, Tokyo, Japan), under bright-field model with 40 × fold magnification. Ap, appressoria; Co, conidia; Gt, germ tubes. Bars = 10 μm.

**Figure 6:**
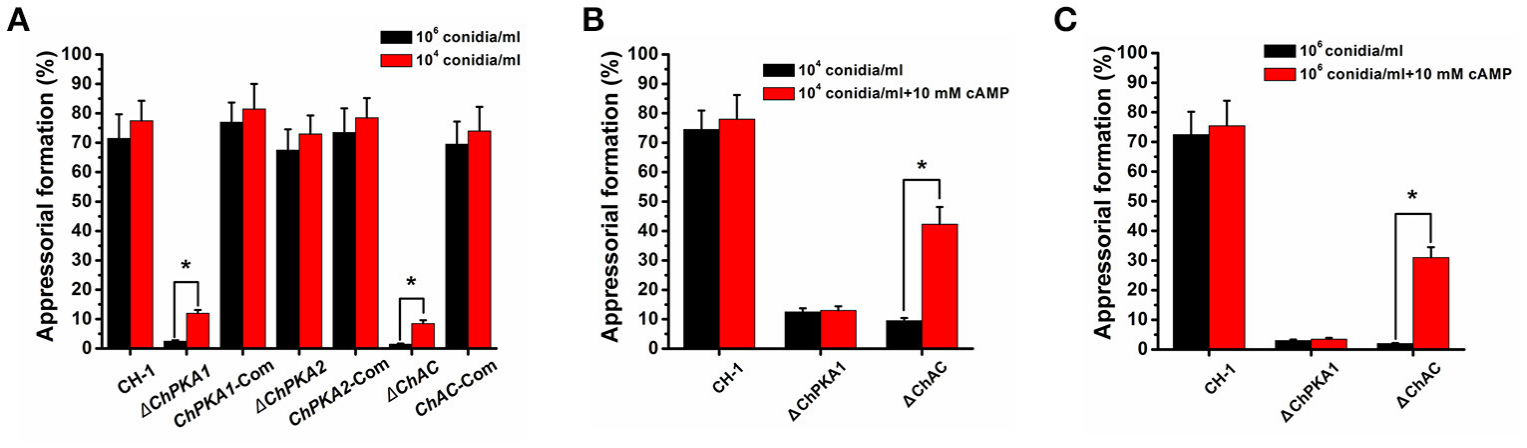
Appressorial formation rate of wild type strain CH-1, Δ*ChPKA1, ChPKA1*-Com, Δ*ChPKA2*, and Δ*ChAC* on hydrophobic coverslips. **(A)** Appressorial formation rate of indicated strains at different conidial density (10^6^ and 10^4^ conidia/ml, respectively) on hydrophobic coverslips at 25°C for 24 h. **(B)** Appressorial formation rate of CH-1, Δ*ChPKA1*, and Δ*ChAC* at 10^4^ conidia/ml with or without exogenous 10 mM cAMP on hydrophobic coverslips at 25°C for 24 h. **(C)** Appressorial formation rate of CH-1, Δ*ChPKA1*, and Δ*ChAC* at 10^6^ conidia/ml with or without exogenous 10 mM cAMP on hydrophobic coverslips at 25°C for 24 h. Asterisks in the graph indicate statistical differences, *P* ≤ 0.01.

The appressorial formation of each strain was also analyzed. The results suggested that at high conidial density (10^6^ conidia/ml), most conidia (>65%) of wild type, complementary strains and Δ*ChPKA2* could form melanized appressoria on hydrophobic surface within 24 h (Figures 5A, 6A). In contrast, most Δ*ChPKA1* and Δ*ChAC* conidia (>97%) did not form appressoria within 24 h. They showed obvious defects in appressorial formation and developed poorly (<3%) on hydrophobic coverslips (Figures 5A, 6A). The appressorium formation rates of Δ*ChPKA1* and Δ*ChAC* were significantly lower than the rate of wild type strain CH-1 and complementary strains (Figure 6A). Interestingly, the appressorial formation defect in Δ*ChPKA1* and Δ*ChAC* could be partially rescued by incubation of conidia at low density (10^4^ conidia/ml), but the appressorial formation rates were still much lower than the rate of wild type strain (Figure 6A). Although conidia of Δ*ChPKA1* and Δ*ChAC* could form appressoria at low density (10^4^ conidia/ml), they developed much longer germ tubes compared with that of the wild type strain CH-1 (Figure 5B). In addition, application of exogenous cAMP could partly restore the size of the germ tube of Δ*ChAC* mutant but not Δ*ChPKA1* (Figure 5B), and exogenous cAMP could also significantly increase the appressorial formation rate in Δ*ChAC* but not Δ*ChPKA1* (Figures 6B,C). These results indicated that *ChPKA1* and *ChAC* play significant roles in regulation of appressorial formation, thus to affect the pathogenicity of *C. higginsianum*.

### cAMP-PKA signaling pathway is involved in stress tolerance

**Figure 7:**
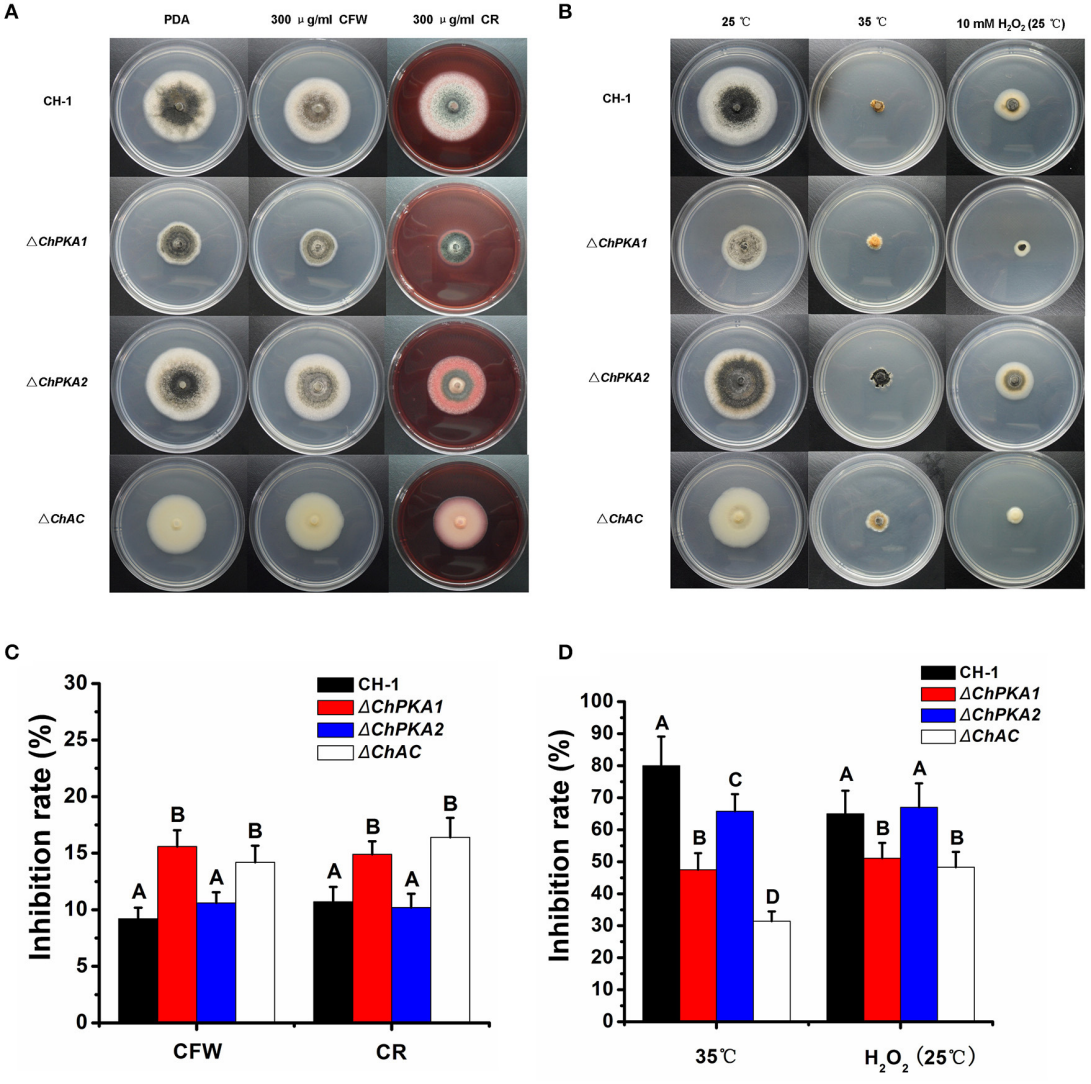
Stress tolerance assays of wild type strain CH-1, Δ*ChPKA1*, Δ*ChPKA2*, and Δ*ChAC*. **(A)** CH-1, Δ*ChPKA1*, Δ*ChPKA2*, and Δ*ChAC* were treated with chemical components in PDA. Images were taken after 6 days of incubation on PDA with 300 μg/ml CFW and 300 μg/ml CR. **(B)** CH-1, Δ*ChPKA1*, Δ*ChPKA2*, and Δ*ChAC* cultured on PDA at different temperature or with 10 mM H_2_O_2_. Images were taken after 6 days of incubation at 25 and 35°C, or with 10 mM H_2_O_2_, respectively. **(C)** Inhibition rate of the radiated growth of CH-1, Δ*ChPKA1*, Δ*ChPKA2*, and Δ*ChAC* on the PDA with chemical components. **(D)** Inhibition rate of the radiated growth of CH-1, Δ*ChPKA1*, Δ*ChPKA2*, and Δ*ChAC* on the PDA at 35°C compared with that of their growth at 25°C or with 10 mM H_2_O_2_. Means and standard deviations were calculated from three replicates. Different letters in the graph indicate statistical differences, *P* ≤ 0.01.

To further examine whether the ChPKA1 and ChAC are involved in stress tolerance, the growth of wild type strain, Δ*ChPKA1*, Δ*ChPKA2*, and Δ*ChAC* were measured on PDA plates supplemented with various compounds including 300 μg/ml Calcofluor White (CFW), 10 mM H_2_O_2_, and 300 μg/ml CR respectively. The tolerance of these strains to high temperature (35°C) was also determined. The results demonstrated that on PDA media with CFW or CR, inhibition of growth rate of Δ*ChPKA1* and Δ*ChAC* was obviously higher than that of wild type strain and Δ*ChPKA2* mutant (Figures 7A,C), indicating that ChPKA1 and ChAC may function to maintain the fungal cell wall integrity by positive regulation.

**Figure 8:**
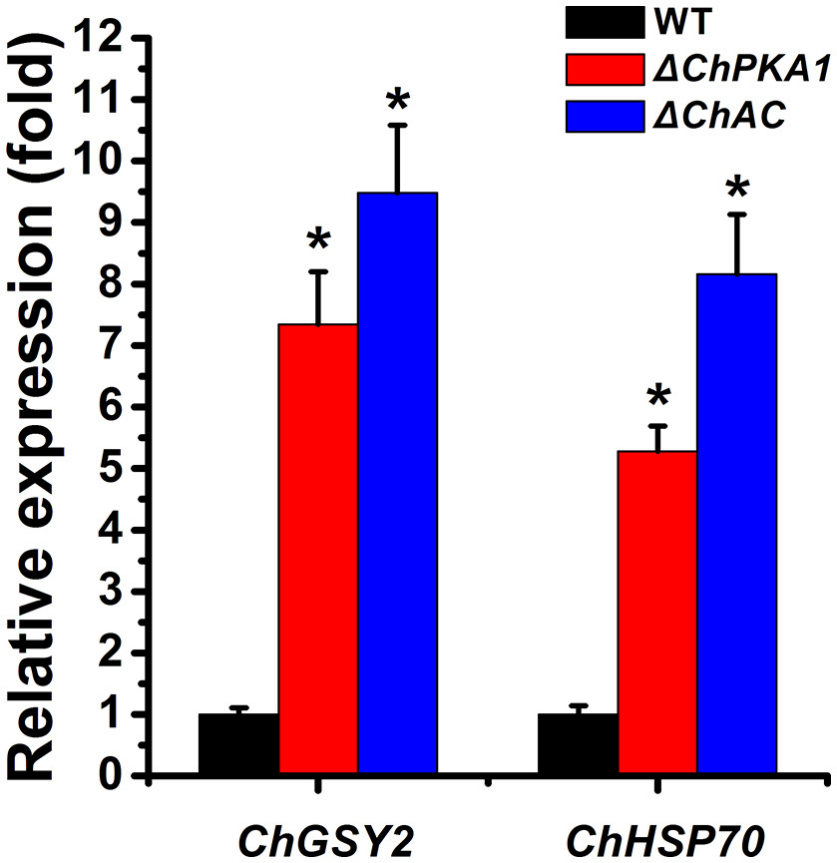
The relative expression of *ChGSY2* and *ChHSP70* in the hyphae of wild type strain CH-1, mutants Δ*ChPKA1* and Δ*ChAC* incubated at 35°C for 3 h. The relative expression of target genes in CH-1 was set as level 1. Expression level of *C. higginsianum* β*-tubulin* (*CH063_04743*) gene was used to normalize different samples. Bars represent standard deviations from three replications. Asterisks indicate statistical differences from Δ*ChPKA1* and Δ*ChAC, P* ≤ 0.01.

In contrast, the Δ*ChPKA1* and Δ*ChAC* mutants showed higher tolerance to 10 mM H_2_O_2_ and high temperature (35°C) compared with wild type strain (Figures 7B,D), which is similar with that of its homolog in *F. verticillioides* (Choi and Xu, 2010), suggesting that ChPKA1 and ChAC may negatively regulate H_2_O_2_ and high temperature tolerance in *C. higginsianum*. Moreover, previous studies reported that the *GSY2* and *HSP26* genes are up-regulated under heat stress condition in yeast (Schnell et al., 1992; Gasch et al., 2000), and the expression levels of *FvGSY2* and *FvHSP26* genes of *F. verticillioides* are higher in adenylate cyclase mutant after heat treatment (Choi and Xu, 2010). Similar results were also reported that the PKA mutant of *F. graminearum* was more tolerant to heat stress than the wild type strain for hyphal growth, and the relative expression levels of *FgHSP70* and *FgGSY2* in PKA mutant were much higher than in wild type strain when cultured at 35°C (Hu et al., 2014). In our study, we speculated that the homolog genes of *GSY2* and *HSP70* in *C. higginsianum* may be also involved in the high temperature tolerance of Δ*ChPKA1* and Δ*ChAC* mutants. In order to verify this assumption, we identified the homolog of GSY2 in *C. higginsianum*, named ChGSY2 (CH063_00792), and homolog of HSP70, named ChHSP70 (CH063_01329). Then, we comparatively analyzed the expression levels of *ChGSY2* and *ChHSP70* in the hyphae of Δ*ChPKA1*, Δ*ChAC*, and wild type strain after incubating at 35°C for 3 h. The result demonstrated that the expression levels of *ChGSY2* and *ChHSP70* were obviously up-regulated in Δ*ChPKA1* and Δ*ChAC* compared with that of the expression in wild type strain (Figure 8), indicating that ChPKA1 and ChAC may negatively regulate the expression of some stress response genes under high temperature condition in *C. higginsianum*.

## Discussion

The cAMP-PKA signaling pathway plays significant and conserved roles in mediating cellular processes, including growth, conidial formation, stress tolerance, and pathogenesis in many fungal pathogens (Turrà et al., 2014; Han et al., 2015; Martin-Urdiroz et al., 2016). However, the roles of cAMP-PKA signaling pathway in anthracnose fungus *C. higginsianum* remain uncharacterized. In this study, we elucidated the functions of ChPKA1, ChPKA2, and ChAC in pathogenic and physiological development of *C. higginsianum*, mainly including growth, conidiation, stress tolerance, appressorial formation, and pathogenicity. Our findings demonstrated that ChPKA1 and ChAC, but not ChPKA2, are essential for growth, appressorial formation, conidiation, and pathogenicity in *C. higginsianum*.

Normally, filamentous fungi have two PKA catalytic subunits genes, but only one of them contributes to the major function of PKA activities. Deletion of the major PKA catalytic subunit gene causes obvious reduction in growth rate and conidiation, whereas the other one plays only a minor role or has no detectable functions (Shimizu and Keller, 2001; Lee et al., 2003; Banno et al., 2005; Ni et al., 2005; Schumacher et al., 2008; Choi and Xu, 2010; Hu et al., 2014). In our study, targeted deletion of *ChPKA1* resulted in significant reduction in growth rate (Figure 3) and conidiation (Figure 4), and sectored colony with obvious dark color (Figure 2A), whereas deletion of *ChPKA2* did not caused any obvious phenotype variation compared with wild type strain. In addition, we also analyzed the phenotype of adenylate cyclase mutant Δ*ChAC*, which showed similar reduction in growth rate (Figure 3) and conidiation (Figure 4). Application of exogenous cAMP could partially rescued the phenotype defects of Δ*ChAC* (Figures 3, 4). These results indicated that ChPKA1 is the major PKA catalytic subunit in the cAMP-PKA signaling pathway of *C. higginsianum*.

In *Aspergillus flavus, F. verticillioides, S. sclerotiorum, F. graminearum, C. lagenarium*, and *F. oxysporum*, deletion of the adenylate cyclase or PKA gene resulted in significant reduction in growth rate (Yamauchi et al., 2004; Jurick and Rollins, 2007; Choi and Xu, 2010; Pei-Bao et al., 2010; Kim et al., 2011; Hu et al., 2014; Yang et al., 2016), whereas the opposite was observed in *M. oryzae* (Mitchell and Dean, 1995; Choi and Dean, 1997; Adachi and Hamer, 1998). In our study, the growth rate of deletion mutants Δ*ChPKA1* and Δ*ChAC* were obviously decreased compared to wild type strain (Figure 3), supporting the diverse roles of cAMP-PKA signaling pathway in different plant fungal pathogens.

Appressorium functions as an important infection structure and plays a critical role in primary penetration The cAMP signaling pathway regulates the appressorial formation in several pathogens. In *M. oryzae*, the feedback between the cAMP-PKA and MAPK signaling pathways regulates appressorium morphogenesis and plant infection (Zhou et al., 2012, 2014). In *C. lagenarium*, cAMP-PKA signaling pathway cooperated with MAPK to regulate appressorial formation and infectious growth (Takano et al., 2000; Yamauchi et al., 2004). Ras GTPase activating protein CoIra1 from *C. orbiculare* was shown to be involved in infection-related morphogenesis and pathogenicity by proper regulation of cAMP and MAPK signaling pathways through CoRas2 (Harata and Kubo, 2014). In our study, deletion of *ChPKA1* and *ChAC* caused obvious defect in appressorial formation at high conidial density (10^6^ conidia/ml). Although defection of appressorial formation in mutants Δ*ChPKA1* and Δ*ChAC* could be partially rescued at low conidial density (10^4^ conidia/ml) (Figures 5A, 6A), conidia of mutants developed much longer germ tubes when compared with that of wild type strain (Figure 5B). Furthermore, the pathogenicity of mutants Δ*ChPKA1* and Δ*ChAC* on *A. thaliana* was also defective (Figure 2B), inferring that defective virulence was possibly caused by the abnormal appressoria formation. In summary, we conclude that cAMP-PKA signaling pathway regulates appressorial formation, thus to affect *C. higginsianum* pathogenicity.

Recently, it was reported that intracellular cAMP level in the vegetative hyphae of *C. gloeosporioides* was controlled by Rho GTPases CgRhoB and regulated conidia germination and stress tolerance (Xu et al., 2016). The Ras guanine-nucleotide exchange factor protein ChRgf acted as an important modulator upstream of several cAMP-PKA signaling pathway to regulate vegetative growth, conidiation, infection-related structure development, and stress responses of *C. higginsianum* (Gu et al., 2017). Our work also examined whether the stress tolerance was affected in cAMP-PKA pathway mutants. The results indicated that the Δ*ChPKA1* and Δ*ChAC* mutants are more sensitive to cell wall inhibitors CFW and CR compared to wild type strain and Δ*ChPKA2* mutant (Figures 7A,C). Interestingly, in contrast, the Δ*ChPKA1* and Δ*ChAC* mutants showed increased tolerance to elevated temperatures and exogenous H_2_O_2_ when compared with wild type strain (Figures 7B,D). In addition, the expression levels of *ChHSP70* and *ChGSY2* in Δ*ChPKA1* and Δ*ChAC* mutants were higher than in wild type strain when cultured at 35°C (Figure 8). These results are similar with previous report that, as well as increased expression level of *HSP70* and *ChGSY2*, the cAMP-PKA pathway mutants are more tolerant to heat stress than wild type strain in *F. verticillioides* and *F. graminearum* (Choi and Xu, 2010; Hu et al., 2014).

Furthermore, in this study, we have also tried several times to generate Δ*ChPKA1ChPKA2* double mutant, but failed, indicating that deletion of both PKA catalytic subunits (*ChPKA1* and *ChPKA2*) is lethal in *C. higginsianum*, which is similar with that of homologs in *A. nidulans* (Ni et al., 2005) and *M. oryzae* (Choi and Xu, 2010). However, this finding is opposite to that of homolog in *F. graminearum* (Hu et al., 2014), suggesting the functional diversity of PKA among diverse fungal species. It also indicated that ChPKA1 and ChPKA2 may have overlapping functions.

Briefly, in this study, we found that cAMP-PKA signaling pathway is involved in appressorium formation, pathogenicity, growth rate, conidiation, and stress tolerance in *C. higginsianum*. Given these properties, targeting cAMP-PKA signaling pathway could be a good strategy to control cruciferous crop anthracnose diseases. The results described above will also enhance our understanding of the interaction mechanisms between *C. higginsianum* and *A. thaliana*.

## Conclusion

ChPKA1 was experimentally confirmed to be the major PKA catalytic subunit in cAMP-PKA signaling pathway and essential to hyphal growth, pathogenicity, appressorial formation, conidiation, and stress tolerance in *C. higginsianum*, indicating that ChPKA1 plays diverse and essential roles in this fungal pathogen.

## Author contributions

Conceived and designed the experiments: WZ and WW. Performed the experiments: WZ and WW. Analyzed the experiment data: WZ, MZ, ZX, FP, and WW. Contributed reagents/materials/analysis tools: WZ, FP, and WW. Wrote the paper: WZ and WW. All authors read and approved the final manuscript.

### Conflict of interest statement

The authors declare that the research was conducted in the absence of any commercial or financial relationships that could be construed as a potential conflict of interest.

## Supplementary material

Supplementary Figure S1 Construction of replacement vectors and confirmation of genes mutants. **(A)** Strategic map of *ChPKA1, ChPKA2*, and *ChAC* replacement construct. The hygromycin phosphotransferase (*hph*) gene cassette was cloned into the corresponding sites to replace the ORFs of *ChPKA1, ChPKA2*, and *ChAC* genes. P1, P2, and P3 indicated the hybridization probes. **(B)** Southern blot analysis of mutants. Genomic DNA (2 μg per lane) of Δ*ChPKA1* and *ChPKA1*-Com were digested with *Kpn*I, genomic DNA (20 μg per lane) of Δ*ChPKA2* and Δ*ChAC* were digested with *Sac*I. The nylon membranes with genomic DNA of *ChPKA1, ChPKA2* and *ChAC* were respectively hybridized with a probe corresponding to P1, P2, and P3. Genomic DNA (20 μg per lane) of wild type strain CH-1 was used as control. **(C)** The relative expression of *ChPKA1* in Δ*ChPKA1* and *ChPKA1*-Com, *ChPKA2* in Δ*ChPKA2*, and *ChAC* in Δ*ChAC*. The relative expression of target genes in CH-1 was set as level 1. Expression level of β*-tubulin* (*CH063_04743*) gene was used to normalize different samples. Bars represent standard deviations from three replications. ND, not detected.

Supplementary Figure S2 Characterization of the *C. higginsianum* ChPKA1 and ChPKA2. **(A)** The domain structures of *C. higginsianum* ChPKA1 and ChPKA2 as annotated by *SMART MODE* (http://smart.embl-heidelberg.de/smart/change_mode.pl). **(B)** Alignment analysis of the amino acid sequences of ChPKA1 and its homologs from other fungi using Clustal X program. MO1: *M. oryzae CPKA* (MGG_06368, E-value: 0.0, 68% identity); FG1: *F. graminearum CPK1* (FGSG_07251, E-value: 0.0, 92% identity); NC1: *Neurospora crassa PKAC-1* (NCU06240, E-value: 0.0, 70% identity); BC1: *Botrytis cinerea PKA1* (BC1G_10410, E-value: 0.0, 68% identity); CL1: *C. lagenarium CPK1* (AB127956.1, E-value: 0.0, 90% identity); VD1: *Verticillium dahliae* (VDAG_06474, E-value: 0.0, 69% identity); SS1: *Sclerotinia sclerotiorum* (SS1G_13577, E-value: 0.0, 68% identity). **(C)** Alignment analysis of the amino acid sequences of ChPKA2 and its homologs from other fungi using Clustal X program. VD2: *V. dahliae* (VDAG_02641, E-value: 0.0, 77% identity); FG2: *F. graminearum CPK2* (FGSG_08729, E-value: 5.2E-171, 77.7% identity); MO2: *M. oryzae CPKB* (MGG_02832, E-value: 0.0, 70% identity); NC2: *N. crassa PKAC-2* (NCU00682, E-value: 0.0, 70% identity); BC2: *B. cinerea PKA2* (BC1G_07542, E-value: 0.0, 74% identity); SS2: *S. sclerotiorum* (SS1G_03171, E-value: 0.0, 72% identity); CG2: *C. gloeosporioides* (AFA28258, E-value: 0.0, 90% identity). **(D)** Phylogenetic analysis of ChPKA1 and ChPKA2 of *C. higginsianum* and their homologs from other fungi. The amino acid sequences were analyzed by MEGA version 4 with Unrooted Neighbor-joining algorithm. Bootstrap values were calculated from 1,000 bootstrap replicates. Only bootstrap support values >50% are shown. The black circle indicates ChPKA1; black triangle indicates ChPKA2.

Supplementary Table S1 Primers used for vector construction and PCR.

Supplementary Table S2 Comparison of growth rate, conidial production, and pathogenicity among mutants, complementary strains and the wild type strain CH-1 of *C. higginsianum*. Means and standard deviations were calculated from three replicates. Different letters in the graph indicate statistical differences, *P* ≤ 0.01.
