# Supplementary figures and images for: The cAMP-PKA Signaling Pathway Regulates Pathogenicity, Hyphal Growth, Appressorial Formation, Conidiation, and Stress Tolerance in Colletotrichum higginsianum
Source: Front Microbiol. 2017 Jul 25;8:1416. doi: 10.3389/fmicb.2017.01416 (PMC5524780; doi:10.3389/fmicb.2017.01416)

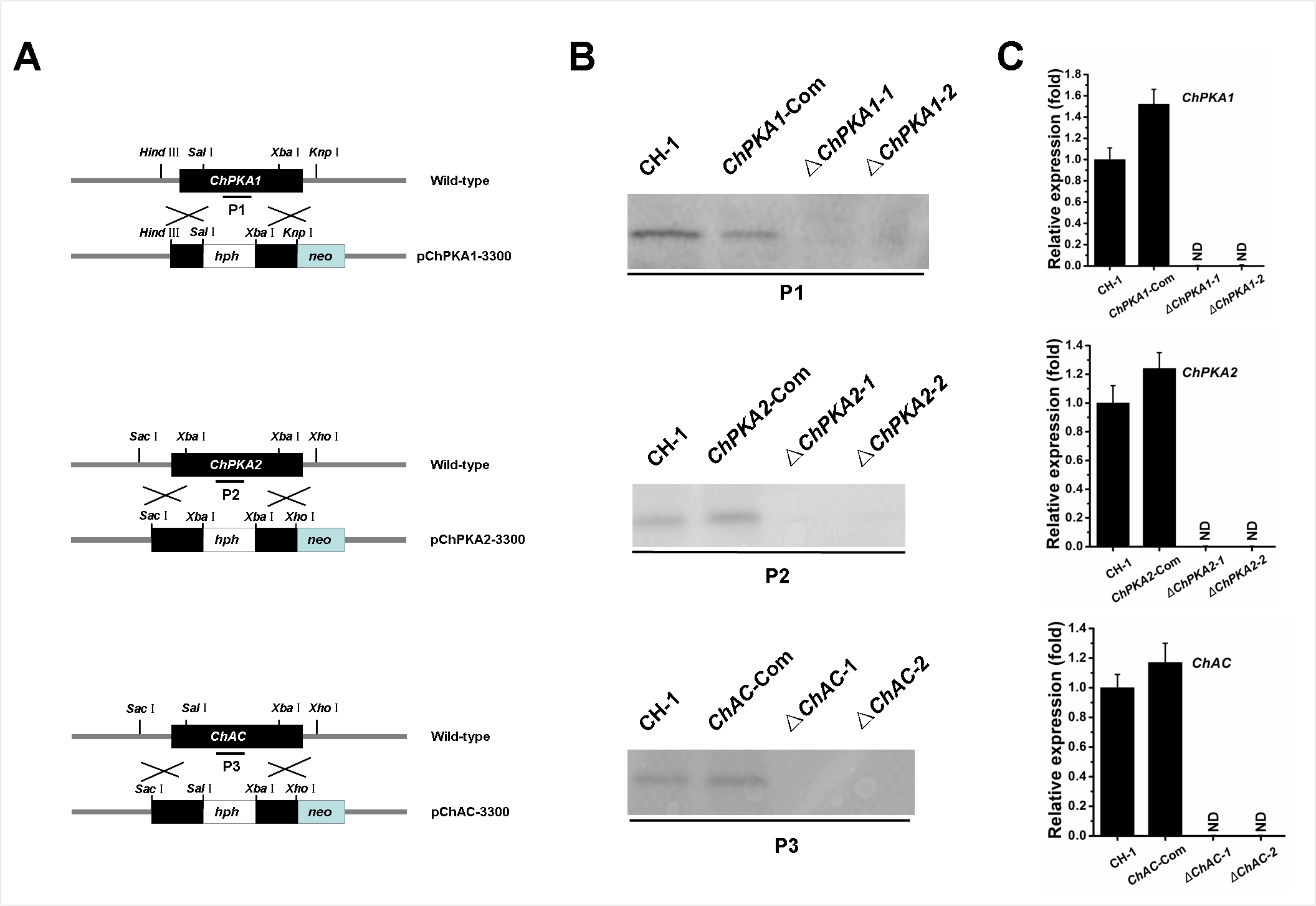

Supplement: Supplementary Figure S1 — Construction of replacement vectors and confirmation of genes mutants. (A) Strategic map of ChPKA1, ChPKA2, and ChAC replacement construct. The hygromycin phosphotransferase (hph) gene cassette was cloned into the corresponding sites to replace the ORFs of ChPKA1, ChPKA2, and ChAC genes. P1, P2, and P3 indicated the hybridization probes. (B) Southern blot analysis of mutants. Genomic DNA (2 μg per lane) of ΔChPKA1 and ChPKA1-Com were digested with KpnI, genomic DNA (20 μg per lane) of ΔChPKA2 and ΔChAC were digested with SacI. The nylon membranes with genomic DNA of ChPKA1, ChPKA2 and ChAC were respectively hybridized with a probe corresponding to P1, P2, and P3. Genomic DNA (20 μg per lane) of wild type strain CH-1 was used as control. (C) The relative expression of ChPKA1 in ΔChPKA1 and ChPKA1-Com, ChPKA2 in ΔChPKA2, and ChAC in ΔChAC. The relative expression of target genes in CH-1 was set as level 1. Expression level of β-tubulin (CH063_04743) gene was used to normalize different samples. Bars represent standard deviations from three replications. ND, not detected. [file Image1.TIF]

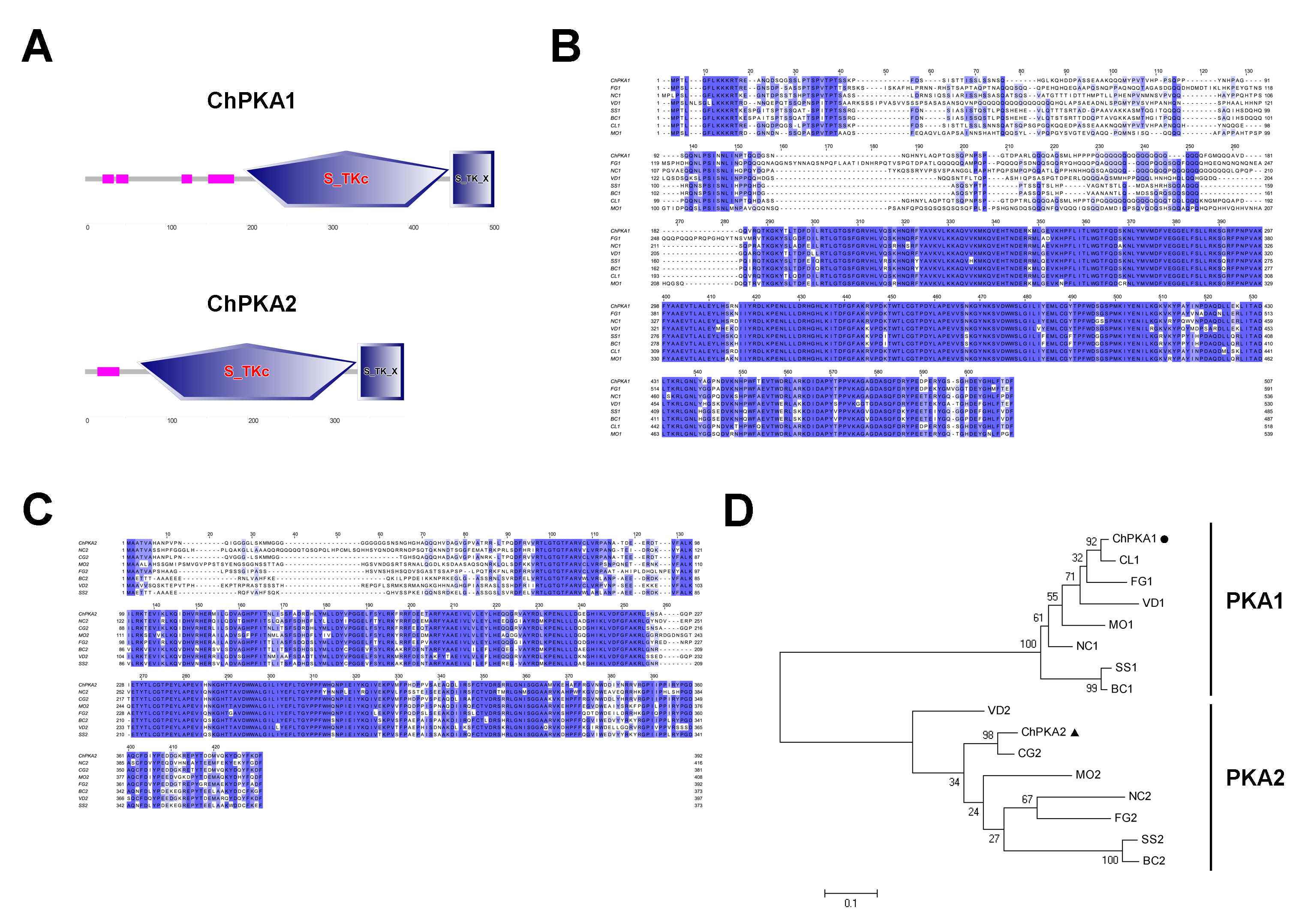

Supplement: Supplementary Figure S2 — Characterization of the C. higginsianum ChPKA1 and ChPKA2. (A) The domain structures of C. higginsianum ChPKA1 and ChPKA2 as annotated by SMART MODE (http://smart.embl-heidelberg.de/smart/change_mode.pl). (B) Alignment analysis of the amino acid sequences of ChPKA1 and its homologs from other fungi using Clustal X program. MO1: M. oryzae CPKA (MGG_06368, E-value: 0.0, 68% identity); FG1: F. graminearum CPK1 (FGSG_07251, E-value: 0.0, 92% identity); NC1: Neurospora crassa PKAC-1 (NCU06240, E-value: 0.0, 70% identity); BC1: Botrytis cinerea PKA1 (BC1G_10410, E-value: 0.0, 68% identity); CL1: C. lagenarium CPK1 (AB127956.1, E-value: 0.0, 90% identity); VD1: Verticillium dahliae (VDAG_06474, E-value: 0.0, 69% identity); SS1: Sclerotinia sclerotiorum (SS1G_13577, E-value: 0.0, 68% identity). (C) Alignment analysis of the amino acid sequences of ChPKA2 and its homologs from other fungi using Clustal X program. VD2: V. dahliae (VDAG_02641, E-value: 0.0, 77% identity); FG2: F. graminearum CPK2 (FGSG_08729, E-value: 5.2E-171, 77.7% identity); MO2: M. oryzae CPKB (MGG_02832, E-value: 0.0, 70% identity); NC2: N. crassa PKAC-2 (NCU00682, E-value: 0.0, 70% identity); BC2: B. cinerea PKA2 (BC1G_07542, E-value: 0.0, 74% identity); SS2: S. sclerotiorum (SS1G_03171, E-value: 0.0, 72% identity); CG2: C. gloeosporioides (AFA28258, E-value: 0.0, 90% identity). (D) Phylogenetic analysis of ChPKA1 and ChPKA2 of C. higginsianum and their homologs from other fungi. The amino acid sequences were analyzed by MEGA version 4 with Unrooted Neighbor-joining algorithm. Bootstrap values were calculated from 1,000 bootstrap replicates. Only bootstrap support values >50% are shown. The black circle indicates ChPKA1; black triangle indicates ChPKA2. [file Image2.TIF]
