# Supplementary material for: The cAMP-PKA Signaling Pathway Regulates Pathogenicity, Hyphal Growth, Appressorial Formation, Conidiation, and Stress Tolerance in Colletotrichum higginsianum
Source: Front Microbiol. 2017 Jul 25;8:1416. doi: 10.3389/fmicb.2017.01416 (PMC5524780; doi:10.3389/fmicb.2017.01416)
Supplement: Supplementary Table S1 — Primers used for vector construction and PCR. [file Table1.DOCX]

**Supplementary Table S1** Primers used for vector construction and PCR.

| **Primer purpose Sequence** | |
| --- | --- |
| *ChPKA1* replacement vector for upstream  *ChPKA1* replacement vector for downstream  *ChPKA2* replacement vector for upstream  *ChPKA2* replacement vector for downstream  *ChAC* replacement vector for upstream  *ChAC* replacement vector for downstream  *ChPKA1* complementary vector | F: 5’ AAGCTTTCTCCCCGTTCTGCTCTCT 3’  R: 5’ GTCGACAGGGTGGTTGTAGGGTGGCT 3’  F: 5’ TCTAGAGGGTTACAACAAATCGGTAGAC 3’  R: 5’ GGTACCGGAGTTAGGAGTGGATAGAGAGG 3’  F: 5’ GAGCTCTCCCAAAACAGTAAAAGCCG 3’  R: 5’ TCTAGAGGGTAAGGGGATTTAGAAGAGAA 3’  F: 5’ TCTAGACAACAGGGGAGGGTCGCATACC 3’  R: 5’ CTCGAGCAACATCTCCGAGCAAACAAC 3’  F: 5’ GGGCCCAGCGATTTCCACTCCGTCA 3’  R: 5’ TCTAGAGCGGCAGAGAGACGAAGTA 3’  F: 5’ TCTAGAGCAGAGGCAAGCGGAACAA 3’  R: 5’ GAGCTCACACGCCACAGAGCCCAGAT 3’  F: 5’ CCATCGATATGCCTACCCTCGGCTTCCTC 3’  R: 5’ AACTGCAGTTAGAAATCGGTGAATAGATG 3’ |
| *ChGSY2* for qRT-PCR  *ChHSP70* for qRT-PCR  *ChPKA1* for qRT-PCR  *ChPKA2* for qRT-PCR  *ChAC* for qRT-PCR  *β-tubulin* for qRT-PCR reference gene  Southern blot probe P1 for *ChPKA1*  Southern blot probe P2 for *ChPKA2*  Southern blot probe P3 for *ChAC* | F: 5’ CCTGCCGAGTGTACCGTCAT 3’  R: 5’ CTGCGATCGACAATGTAGATGC 3’  F: 5’ TCACCCTGAACCAAGACGAGG 3’  R: 5’ AACTCGATGGGGTAGCTCATG 3’  F: 5’ CACACCAATGACGAGAGGAAGAT 3’  R: 5’ TAGAATTTCGCGACAGGGTTAGG 3’  F: 5’ GCTGTTCAGTTACCTGCGCAAG 3’  R: 5’ AGGAGGTTCTCGGGCTTGAG 3’  F: 5’ CGACTGGAACTGGAACCTCAAC 3’  R: 5’ GTGACATCCATGAGGCCTATGAC 3’  F: 5’ AGAAAGCCTTGCGACGGAACA 3’  R: 5’ CCTCCAGGGTTTCCAGATTA 3’  F: 5’ GCCATAACTACCTAGCCCAGC 3’  R: 5’ TCAACAAAGTCCATCACCATGT 3’  F: 5’ AGCAGCATGTCGACGCTGG 3’  R: 5’ CTCGTGCAGATACTCGAGCACC 3’  F: 5’ AACCTGCCTGCCGATATATCC 3’  R: 5’ AAGATGTGACCAAAGTTCTCGTG 3’ |
