# Supplementary material for: The cAMP-PKA Signaling Pathway Regulates Pathogenicity, Hyphal Growth, Appressorial Formation, Conidiation, and Stress Tolerance in Colletotrichum higginsianum
Source: Front Microbiol. 2017 Jul 25;8:1416. doi: 10.3389/fmicb.2017.01416 (PMC5524780; doi:10.3389/fmicb.2017.01416)
Supplement: Supplementary Table S2 — Comparison of growth rate, conidial production, and pathogenicity among mutants, complementary strains and the wild type strain CH-1 of C. higginsianum. Means and standard deviations were calculated from three replicates. Different letters in the graph indicate statistical differences, P ≤ 0.01. [file Table2.DOCX]

**Supplementary Table S2** Comparison of growth rate, conidial production and pathogenicity among mutants, complementary strains and the wild-type strain CH-1 of *C. higginsianum*. Means and standard deviations were calculated from three replicates. Different letters in the graph indicate statistical differences, P ≤ 0.01.

| **Strains** | **Growth rate (cm/d)** | | **Conidiation**  **(×10^5^ conidia/plate)** | **Pathogenicity** |
| --- | --- | --- | --- | --- |
| CH-1  *ΔChPKA1-1*  *ΔChPKA1-2*  *ΔChPKA1-3*  *ΔChPKA1-4*  *ΔChPKA1-5*  *ChPKA1*-Com-1  *ChPKA1*-Com-2  *ChPKA1*-Com-3  ΔChPKA2-1  ΔChPKA2-2  ΔChPKA2-3  ΔChPKA2-4  ΔChPKA2-5  *ChPKA2*-Com-1  *ChPKA2*-Com-2  *ChPKA2*-Com-3  ΔChAC-1  ΔChAC-2  ΔChAC-3  ΔChAC-4  ΔChAC-5  *ChAC*-Com-1  *ChAC*-Com-2  *ChAC*-Com-3 | 0.44±0.04^A^  0.22±0.01^B^  0.23±0.02^B^  0.23±0.02^B^  0.24±0.01^B^  0.21±0.03^B^  0.45±0.03^A^  0.46±0.04^A^  0.43±0.02^A^  0.41±0.03^A^  0.42±0.02^A^  0.42±0.02^A^  0.44±0.03^A^  0.43±0.01^A^  0.43±0.03^A^  0.41±0.01^A^  0.44±0.03^A^  0.33±0.01^C^  0.32±0.02^C^  0.31±0.01^C^  0.34±0.03^C^  0.33±0.03^C^  0.44±0.05^A^  0.45±0.02^A^  0.43±0.03^A^ |  | 8.5±0.75^A^  4.1±0.55^B^  4.2±0.35^B^  3.9±0.40^B^  4.5±0.65^B^  4.0±0.35^B^  7.9±0.83^A^  8.2±0.77^A^  8.1±0.68^A^  8.9±0.64^A^  8.4±0.57^A^  8.3±0.87^A^  7.9±0.82^A^  8.6±0.79^A^  8.2±0.89^A^  8.6±0.84^A^  8.0±0.82^A^  1.2±0.21^C^  1.1±0.13^C^  1.4±0.19^C^  1.0±0.09^C^  1.1±0.15^C^  8.1±0.78^A^  8.3±0.68^A^  7.9±0.81^A^ | Yes  No  No  No  No  No  Yes  Yes  Yes  Yes  Yes  Yes  Yes  Yes  Yes  Yes  Yes  No  No  No  No  No  Yes  Yes  Yes |
